# Process-oriented evaluation of an international faculty development program for Asian developing countries: a qualitative study

**DOI:** 10.1186/s12909-017-1101-2

**Published:** 2017-12-21

**Authors:** Do-Hwan Kim, Jong-Hyuk Lee, Jean Park, Jwa-Seop Shin

**Affiliations:** 1 Department of Medical Education, Seoul National University College of Medicine, 103 Daehak-ro, Jongno-gu, Seoul, 03080 Republic of Korea; 2 Department of Medical Education, Eulji University School of Medicine, 77 Gyeryong-ro 771 beon-gil, Jung-gu, Daejeon, 34824 Republic of Korea; 3 WHO Collaborating Centre for Educational Development, Seoul National University College of Medicine, 71, Ihwajang-gil, Jongno-gu, Seoul, 03087 Republic of Korea

**Keywords:** Developing countries, Faculty development program, Health professions education, Southeast Asia, Thematic analysis

## Abstract

**Background:**

Non-English-speaking developing countries in Southeast Asia have been provided only limited opportunities for faculty development in the education of health professions. Although there exist a few programs that have been shown to be effective, they are frequently presented with few explanations on how and why the programs work due to their outcome-oriented nature. This study explores the process of the Lee Jong-Wook Fellowship for Health Professional Education, an international faculty development program designed for capacity building of educators of health professions in Southeast Asian developing countries.

**Methods:**

Fellows were from Cambodia, Myanmar, and Laos. Qualitative data were collected from two types of semi-structured interviews – group and individual. Thematic analysis was conducted to explore the factors related to the effectiveness of the program, framed by four components of faculty development, which included context, facilitators, program, and participants.

**Results:**

From the thematic analysis, the authors identified a total of 12 themes in the four components of faculty development. In the context domain, the resource-poor setting, a culture that puts emphasis on hierarchy and seniority, and educational environment depending on individual commitment rather than broad consensus emerged as key factors. In the facilitators domain, their teaching methods and materials, mutual understanding between teacher and learner, and collaboration between facilitators mainly influenced the learning during the fellowship. In the program domain, the key advantages of the fellowship program were its applicability to the workplace of the fellows and enough allowed time for practice and reflection. Finally, in the participants domain, Fellows valued their heterogeneity of composition and recognized cognitive as well as non-cognitive attributes of the participants as essential.

**Conclusions:**

This process-oriented evaluation reveals the diverse factors that contributed to achieving the intended outcomes of the fellowship. Although much evidence from best practices in faculty development are still valid, the findings suggest that the selection strategies, learning environment, and English communication should be given more consideration when organizing a program targeting these people and cultures. A comprehensive understanding of the process would contribute to developing tailored strategies for educators of health professions in developing countries in similar settings.

**Electronic supplementary material:**

The online version of this article (10.1186/s12909-017-1101-2) contains supplementary material, which is available to authorized users.

## Background

Strengthening the educational system can improve the performance of health professionals and ultimately contribute to the improvement of population health [1]. One of the most common types of reported educational interventions is a faculty development program (FDP), which can have varying formats including workshops, short courses, seminar series, and longitudinal interventions [2]. Previous studies have reported that FDPs generally have positive outcomes across satisfaction, learning, and behavioral changes, regardless of their formats [2–4]. Provided that faculty development was described as an imperative rather than a luxury [5], supporting the training of educators of health professionals therefore is a necessary intervention in developing as well as developed countries [6, 7].

Nonetheless, in practice, FDPs in the field of health professions education (HPE) or medical education are mostly conducted by advanced, English-speaking countries, and at institutional- or national- levels [2, 8]. To paraphrase, educators in health professions in many developing regions have not been provided with sufficient opportunities to participate in FDPs to build their educational capacity [9], and Southeast Asia is one of them. The situation might be attributable to several region-specific issues that need to be considered when planning and implementing international FDPs for Southeast Asian countries. First, these countries are culturally and geographically distant from North America and Western Europe, the current mainstream of HPE [10]. These cultural differences might be the cause of decreased compatibility and effectiveness of educational approaches rooted in a Western context [11]. Second is the language issue, which originates from the fact that many Southeast Asian countries are non-English-speaking countries. Concerning the “brain-gain” of health personnel, it has been reported that their non-fluent English acts as a critical barrier, not only when they apply for overseas programs but also when outside trainers visit and conduct trainings [6, 12]. Third, collaboration for faculty development is lacking even among Southeast Asian countries [13]. As a consequence, faculty development activities within this region have been conducted disproportionately with Cambodia, Laos, and Myanmar only accounting for 6% of the reported activities [13]. What is interesting is that, paradoxically, the peripheral status of these countries often provided faculty developers with novel opportunities to establish international faculty development partnerships and to train health professionals. For example, Kim et al. developed an international FDP called the Seoul Intensive Course for Medical Educators (SICME) in collaboration with five Asian developing countries and demonstrated the outcome in terms of the participants’ reaction, learning, and behavior [14].

In addition to these issues related to implementation, the existing literature is limited by its primary focus on evaluating outcomes. Although it is important, demonstration of outcomes is not sufficient to deepen our understanding of faculty development in developing countries because mere evidence on whether the program achieved the intended outcome does not provide proper explanations on how and why the program worked [15]. Additionally, note that the “process” itself is an essential component that defines the success of a partnership, especially in the case of FDPs based on international collaboration [16]. Therefore, conventional outcome-oriented research on international FDPs need to be complemented by expanding process-oriented studies using qualitative methods [3]. This is also consistent with previous calls for more clarification research that focuses on “the processes that underlie observed effects” using a conceptual framework [17].

In 2011, a useful conceptual framework for research on faculty development was suggested by O’Sullivan and Irby [18]. The authors criticized the causal assumption underlying the traditional, linear, outcome-oriented model and proposed to embed faculty development into two communities – a workplace community and faculty development community – which share four primary, process-related components – context, facilitator, program, and participants. Although Plack et al. pointed out this framework does not explain “how” those components actually operate in faculty development [19], several studies were conducted based on the framework to explore social or contextual factors affecting the outcome such as a change in teaching practice or organizational impact [19, 20]. However, regarding studies on international faculty development partnerships with Southeast Asian countries, let alone explicitly stating any framework to contextualize the findings, only a few have dealt with factors that influence the process; for example, a study on a training program for nurses in Cambodia identified major barriers such as an inadequate environment and difficulties in colleague relationships [21] and another study in Laos argued that factors such as learning materials, instructors, and clinical environment affected effectiveness of a physician training program [22]. In fact, most of these factors could be associated with the components in O’Sullivan and Irby’s framework. That is, by employing the framework, it is expected to draw out implications more comprehensively with regard to capacity building of health professions in developing countries.

The main research question of this study is as follows: what factors impact on the outcomes of the LJWF-HPE, which was designed for capacity building of health professions educators in non-English-speaking Southeast Asian developing countries, in terms of the four primary components suggested by O’Sullivan and Irby? Specifically, we sought to understand the perspective of the fellows in training during the program. The implications for the development and implementation of the international FDP targeting non-English-speaking developing countries are also discussed.

## Methods

### Design

We conducted a qualitative study using semi-structured interviews with eight fellows who participated in the LJWF-HPE. Because the existing literature on process-oriented evaluation of international FDPs for developing countries in Southeast Asia is not sufficient, we used thematic analysis to inductively explore how the four primary components of faculty development – context, facilitator, program, and participants – influenced the effectiveness of the program. Individual interviews as well as group interviews were used because each method has distinct advantages which are mutually complementary. For example, group interview enables participants to react to and build on the ideas of others; however, it is less suitable to gather sensitive or personal information than that of individual interviews [23]. Although the interview agendas were not strictly divided according to the types of interviews, any personal topic that could be delicate was mainly assigned to individual interviews.

### Context and participants

In 2014, Seoul National University College of Medicine implemented an international FDP called the SICME. The SICME has focused on Asian developing countries in which existing programs are marginalized, and the outcome evaluation based on Kirkpatrick’s four-level model has confirmed its effectiveness regarding level 1 to level 3 of the model [14]. In recognition of these achievements, since 2016, SICME has been given funding by the Korea Foundation for International Healthcare (KOFIH), a government-funded institute which is planned to be sustained as the Lee Jong-Wook Fellowship for Health Professional Education (LJWF-HPE).

The LJWF-HPE was held from October 17 to December 9, 2016. Comparing the LWJF-HPE with other international fellowship programs, such as the Harvard Macy Institute Program [24] or the FAIMER Institute [7], the most noticeable difference is how the sessions were arranged. While others made residential sessions interspersed by months to provide time to work on one’s educational project at his/her own institution, the LJWF-HPE provided entire courses over eight consecutive weeks outside of the fellows’ workplace.

The LJWF-HPE, as a follow-up to the SICME whose details are described in Kim DH et al. [14], for the most part kept the central initiatives of the SICME - multi-level collaboration and the four design principles - the same. However, there have been minor changes in the details because the KOFIH took the role of the funding agency. When compared with the SICME, the total training period increased from 6 to 8 weeks, while the total training time increased from 150 to 165 h (Table 1). The number of participating facilitators decreased from 23 to 21, and the number of participating countries decreased from five to three (Cambodia, Laos, and Myanmar) excluding Vietnam and Mongolia. The total number of trainees decreased from 16 to 8. For Cambodia and Myanmar, to select the fellows, the requirements for eligible participants were given to the respective embassies, and they were called as well for recommendations for appropriate candidates. The eligibility criteria include education (major in health profession discipline), age (<56 years old), physical and mental status, work experience, and English proficiency. As a project manager (PM), one of the authors (JSS) then conducted a one on one interview using Skype and selected the final fellows. The Laotian fellow was selected from the Lee Jong-Wook Seoul Project, a separate fellowship of the KOFIH [25], and participated in the LJWF-HPE on behalf of a recommendation from the PM with the consent of the KOFIH.

**Table 1:** Overview of the LJWF-HPE

Week	Modules and Topics	Total hours of education	Number of facilitators involved
1st week	Module 1: Theory & Practice of Teaching and Learning	25	4
2nd week	Module 2: Student Selection and Admissions	5^a^	2
3rd week	Module 3: Curriculum Development and Evaluation	25	3
4th week	Module 4: Student Assessment	30^b^	4
5th week	Module 5: Educational Administration & Project Management	30^c^	4
6th week	Module 6: Technology in Medical Education	10	4
6th week	Module 7: Human Resource for Health Policy	10	2
7th week	Module 8: National Licensing Examination	7	2
7th week	Module 9: Accreditation	8	3
8th week	Module 10: ODA Design and Development for Human Resource for Health	15	1
Total		165	21^d^

^a^All fellows participated the Asia-Pacific Joint Conference on PBL 2016 for three days during this week; ^b^One supplementary session was added on the 6th week by the request of fellows; ^c^One day in the 8th week was allotted for this module; ^d^Five facilitators participated in more than two modules

*LJWF-HPE* Lee Jong-Wook Fellowship for Health Professional Education *ODA* Official Developmental Assistance

### Interview

#### Instrument

The first author drafted a guide for semi-structured interviews in English. It is comprised of three sections: (1) Before fellowship, (2) During fellowship, and (3) After fellowship (see Additional file 1). Each question had its primary use either for group interviews or individual interviews, but fellows were not prevented from mentioning any other topics when they occurred spontaneously in the interview process. Minor edits were made to the draft through a review by the project assistants and the PM.

#### Procedure

The interview team consisted of three of the authors (DHK, JHL, and JP), who had a role as a teaching assistant or project assistant in the LJWF-HPE. Their primary responsibilities included preparing the teaching materials when required by the facilitators and being in the class for any ad hoc requests but did not teach or assess fellows directly. Concerned with the influence from potential power relations, JSS, who not only participated as a facilitator but also interviewed and selected the fellows by himself as the PM, did not engage in the data collection.

All interviews were conducted by the first author (DHK) to ensure consistency. In the group interviews, JHL or JP participated to observe and document the nonverbal interaction and group dynamics. We were aware that Asians are concerned with loss of face more often than not [11] and that social desirability bias might make answers be positively biased in this type of evaluation [26]. Therefore, when nonverbal signs such as being hesitant or remaining silent were observed, we cautiously probed for more details and candid responses. In addition, as Lingred et al. states, the formation of a rapport between researchers and participants is key to maximizing the authenticity of the collected data [27], the interviews started when the fellowship was halfway through rather than from the beginning.

Sufficient information was provided to all participants, and consent was obtained verbally. There was no incentive for participating in the interviews. To relieve the fellows’ potential feeling of obligation, we ensured that decision would remain completely voluntary. We also stressed that raw data will be accessible to only three of the authors who engaged in the data collection firsthand. Furthermore, it was assured that the purpose of interview will be confined to the evaluation of the LJWF-HPE and refinement of the following program, and it has nothing to do with the assessment of individual trainees. All fellows agreed to participate in ten group interviews (one for each module), and seven fellows, except for one who submitted written responses as an alternative, agreed to participate in individual interviews. Data collection and analysis were performed concurrently, and although data saturation was reached before all the interviews were completed, all planned group and individual interviews were conducted because the interviews were included as part of the LJWF-HPE. Each interview lasted 40 to 70 min.

Because the fellows were non-native speakers of English, several actions needed to be taken to foster sufficient reflection and free flow of expression. First, one week before starting the actual interviews, a meeting was held with all the fellows to review the interview questions. Here, the fellows could clarify the meaning of each question, then arrange their schedule taking into consideration the time required to prepare for the interviews. Second, during the interviews, they were encouraged to search dictionaries or the internet as needed. Lastly, while individual interviews were conducted on a one-on-one basis to obtain truthful answers, a few participants requested to have a close fellow present in the interview to seek help for clearer English communication. The request was accepted if both fellows agreed.

### Data analysis

#### Thematic analysis

Data was analyzed using an inductive thematic analysis [28] framed by four primary components of faculty development research suggested by O’Sullivan & Irby [18] as the highest-level categories. All interviews were audio recorded, and DHK transcribed verbatim immediately after the interviews. All three authors who participated in the data analysis (DHK, JHL, and JP) were responsible for assisting staff during the fellowship process. By virtue of being an insider, we could avoid superficial analysis and seek a more nuanced interpretation of the interviews which were about a specific context such as modules, topics, or facilitators.

The thematic analysis was conducted in six steps. In the first step, the three authors checked the accuracy of all initial transcripts and repeatedly read to familiarize themselves with the data. Next, each author independently generated initial codes for what they considered meaningful. In steps three and four, themes were identified and reviewed, respectively. In these stages, three authors gathered to present and combine the initial codes to derive candidate themes. The authors resolved discrepancies through iterative discussions until a consensus was reached. When a new theme emerged, it was constantly compared with existing themes. Once the thematic map was prepared, themes were defined, refined, and named to best represent the codes included. In the sixth step, quotes for each theme to be included in the report were selected.

#### Trustworthiness

To enhance the trustworthiness of the study, the researchers used various strategies according to the four criteria suggested by Lincoln and Guba [29]. To ensure the credibility of the data collection process, the researchers conducted two different types of interviews for methods triangulation. In the data analysis, all researchers participated in investigator triangulation, and the fellows were asked to review and give comments to the final draft of the analysis (member check). A detailed description of the study setting was included to improve the transferability, and the results were compared to those from the other research settings. The three professional educators who did not participate in the study audited the steps and decisions made during the analysis, including documentation of the memos, thematic analysis process, and choice of illustrative quotes, to ensure dependability. Finally, minority as well as majority opinions were included to ensure the confirmability of the research findings.

## Results

Of the eight fellows who agreed to participate, four (50%) were male. Four of the fellows were from Myanmar, three from Cambodia, and one from Laos. The number of participants who majored in medicine, pharmacy, and nursing was three, three, and two, respectively. The participants were primarily faculty members of various universities (*n* = 6; 75%), while the remaining two were government officials from the Ministry of Health in Myanmar.

### Context

In this domain, themes that characterize the socio-cultural environment to which fellows belong were identified, which includes a lack of resources, cultural background, and educational environment (Table 2).

**Table 2:** Themes and illustrative quotes for the context domain^a^

Themes	Sub-themes	Quotes
Lack of resources		*It’s still far from our ability to apply these technologies in our current situation. We will need IT technicians, good internet connection, and more computers. Also, regarding high fidelity simulators, there are some maintenance problems. (Fellow E, MOH, Medicine)*
		*Most of the teachers who come to teach at UHS, they have their own private clinics to make more money. Most of the time, they do not want to attend (a workshop or fellowship) unless any incentives are given. However, we cannot ignore them, because we really need those people. We don’t have other resources. (Fellow D, University, Nursing)*
Cultural background	Obedience to hierarchy	*Promotion, no… But I might be involved in educational work. It depends on the decision of the superiors. But, they did not tell me and usually never tell us before (the decision is made). (Fellow H, MOH, Medicine)*
		*I have authority and I can call my subordinates to my office and tell them ‘let’s do this’. I can arrange (projects). (Fellow C, University, Nursing)*
	Respect for seniority	*So, if I join the training, the trainees will be my teachers who taught me in my university years, because I graduated that university as well. So, some of them would say, “You were my student. How can you come teach me how to teach? I taught you well until you become like today”. That’s what concerns me the most. (Fellow A, University, Pharmacy)*
Educational environment	Lack of consensus among faculty members in workplace	*Terminology like “outcome-based curriculum” is already known, but we don’t actually have agreement on what it is. Different faculty members have different conception or understanding regarding terminologies and it delays the process (Fellow B, University, Medicine)*
	Dependency on personal commitment	*There is no institutional encouragement in applying new teaching methods. Whether you do it or not, there is no difference including recognition from dean or colleagues. However, students like it (the changes) and I could feel it. (Fellow D, University, Nursing)*
		*I plan to send a report in person to the rector and dean which covers brief explanation of important points. I hope this may increase the chance of application (of the knowledge gained). I myself always put the collective interest first. Whenever the school needs me, I will gladly share my experience. (Fellow F, University, Pharmacy)*

^a^For each quote, the type of affiliated institution and professional background are provided in the parentheses

*UHS* University of Health Sciences *MOH* Ministry of Health

### Lack of resources

The most frequent phrase that fellows used to describe their current situation was “under-resourced” or “limited resources,” which included lack of finances, facilities, and human resources. Fellows were concerned that applicability and utility of some of the contents of the fellowship would decrease due to the restriction of available resources to be invested in educational innovation. More importantly, one commonly observed consequence of a resource-poor setting was faculty members who run private clinics to compensate for a low salary. Given that many faculty members earn a higher income by spending their time outside the university, it becomes even more challenging to encourage participation in FDPs which provide little incentives.

### Cultural background

#### Obedience to hierarchy

Many fellows mentioned about the tendency of accepting power distance and following directions from a person higher in rank even when it seemed unreasonable. Within the cultural context of Southeast Asia, the instructions from hierarchy were difficult to resist for the subordinates. Likewise, concerning education, fellows referred to the intention of their supervisor as a key factor in determining how much they would be able to apply their learning after returning to their institutions. However, a few said that this situation is not always disadvantageous especially when fellows possess the higher position in the organizational hierarchy. In that case, inducing the intended changes in education was expected to be more feasible.

#### Respect for seniority

In addition to the organizational structure that appreciates hierarchy, some fellows expressed a sense of pressure from a culture that respects seniority, which will be an impediment to effective functioning as a faculty developer. For example, senior faculty members often tend to adhere to the existing education style. Due to cultural characteristics, not only do fellows who are relatively younger in age feel uncomfortable to plainly argue the necessity of change, but also seniors may perceive such an attitude to be inappropriate or even rude.

### Educational environment

#### Lack of consensus among faculty members in workplace

Fellows pointed out the difficulty of drawing consensus among faculty members as a typical problem that prevents effective educational change. In an institution, despite a strong initiative to reform the current time-based curriculum into a competency-based curriculum, it was particularly demanding to reach a broad consensus on core concepts, and eventually, the change process slowed down.

### Dependency on personal commitment

The comments of the fellows revealed the heavy dependence of the institutions on the willingness and motivation of individual faculty members to improve teaching practices. Fellows noted that even though students respond positively when new teaching methods are adopted, their institutions usually lack policies or incentives to further induce or sustain the educational improvement. Likewise, fellows noted their personal interest and enthusiasm rather than institutional support were crucial in the transfer of learning although they participated in the LJWF-HPE on the recommendation of the institution.

### Facilitators

Twenty-one facilitators from nine institutions participated in the LJWF-HPE. In this domain, the analysis showed that the teaching methods and materials, mutual understanding between the teacher and learner, and collaboration between facilitators were the major influencing factors (Table 3).

**Table 3:** Themes and illustrative quotes for the facilitators domain^a^

Themes	Quotes
Teaching methods and utilization of materials	*Facilitator A facilitated well in the debate section to involve all participants in the debate. Also, the debate followed by a brief summary of the facts based on literature was interesting and helpful. (Fellow B, University, Medicine)*
	*Sometimes I face hardships in following lessons during the class, but it is not critical because we can see what the facilitators want to give through handouts. (Fellow H, MOH, Medicine)*
	*Actually, the facilitator gave those (lecture materials) in advance, but he did not give any instruction. So most of us did not think that we should have read those materials before the class (Fellow E, MOH, Medicine)*
Mutual understanding between teacher and learner	*Facilitators were more impressive when they seemed to well understand our situation of developing countries (Fellow B, University, Medicine)*
	*Even though facilitator B speaks English fluently, sometimes it was not very understandable. In contrast, facilitator C’s English was more clear, short, and easy to understand (Fellow D, University, Nursing)*
	*When we meet a facilitator for the first time, sometimes it is difficult to understand what he/she says. But as we spend more time with him/her throughout the program, we adapt to the teaching style and understand more easily about what the facilitator says (Fellow E, MOH, Medicine)*
Collaboration between facilitators	*They (facilitators) had really good coordination, and complemented each other. Especially during the discussion, two professors worked together to actively facilitate us. (Fellow F, University, Pharmacy)*
	*During fellowship, I learned various styles of facilitating participants while experiencing dozens of facilitators. It would help me a lot in my teaching. (Fellow E, MOH, Medicine)*
	*The quality of contents delivered by facilitator D depended on who was in charge of the translation. When I felt that the interpreter subjectively changed what facilitator D initially meant, it made me less convincing about the contents. (Fellow A, University, Pharmacy)*

^a^For each quote, the type of affiliated institution and professional background are provided in the parentheses

*MOH* Ministry of Health

### Teaching methods and utilization of materials

Fellows recognized that most facilitators maintained a learner-centered, participatory approach during the whole course of the program. In respect to the teaching methods, the use of various learning activities with didactic lectures at an appropriate ratio also contributed to effective learning. Timely feedback from facilitators helped the fellows to check whether they were on the right track.

The provided learning materials were particularly useful when the fellows tried to catch up on lectures about contents they did not fully understand in class. For soft copies, several fellows considered using the given lecture slides directly as educational materials in their institutional FDPs unless there was a copyright infringement. However, some fellows reported that the perceived utility of the materials was considerably degraded, especially when they felt overwhelmed by the amount of materials or there was no clear guidance on how to use them.

### Mutual understanding between teacher and learner

Most of the fellows said their learning as well as satisfaction improved when the instructors were well aware of the fellows’ situation. Because they shared similar experiences as health professionals and educators working in developing countries, facilitators who took these commonalities into account were acknowledged to maintain the relevance of the education. On the other hand, it was also important to consider the variability of the trainees, especially for ‘background knowledge’ and ‘English proficiency.’ Most fellows mentioned that in general, facilitators whose English expressions were concise and understandable, although maybe not fluent, were more helpful.

For several fellows, it was meaningful to meet the same instructor repeatedly during the program. When facilitators participated in more than one module and taught them various topics, fellows could adapt to that facilitator’s teaching style, and the teaching and learning process became more efficient.

### Collaboration between facilitators

Because usually two or more facilitators were involved in a single module, the degree of coordination and cooperation among them was found to affect the overall educational effectiveness. Having multiple facilitators was most appreciated when sessions were organized in a way to complement each facilitator’s teaching styles and perspectives. Moreover, many fellows mentioned that experiencing twenty-one different facilitators provided opportunities to compare and attain proper attitudes and facilitation skills needed as faculty developers, yet there was no formal session for training the facilitation skill. For these reasons, the fellows generally preferred to be taught by multiple instructors even if the coordination was somewhat suboptimal. However, for some facilitators who were accompanied by an interpreter, the reliability of the content itself was threatened if the fellows felt that the interpreter could not accurately convey the original meaning.

### Program

#### Applicability to the workplace

One of the most frequently mentioned strengths of the fellowship was that it dealt with topics applicable to the actual duties in the workplace (Table 4). Because all participants were responsible for all kinds of teaching in HPE, most of the contents were considered appropriate in terms of relevance and practicality. In addition to the contents, the products that the fellows developed as individual assignments or learning tasks were expected to be readily implementable when they return to their countries.

**Table 4:** Themes and illustrative quotes for the program domain^a^

Themes	Quotes
Applicability to the workplace	*In module 3, curriculum development and evaluation, each of us had presentation on one’s own plan. Our university has a plan to change the curriculum into competency-based curriculum, and I’m sure that it (my plan) could be applied directly after this fellowship. (Fellow F, University, Pharmacy)*
Provide enough time for in-class application and after-class reflection	*Why I am happy in here is that I can actually practice what I learned in each session. Compared to the similar courses I had in Vietnam, Thailand, and Hong Kong, this program is more organized to focus on application, which made my idea clearer. In addition, reflecting on my training experience before, a fellowship with eight hours of education per day imposed a lot of homework. However, I am very happy with this fellowship because now I have time to do some self-directed learning after class. (Fellow F, University, Pharmacy)*
Selection of fellows	*I don’t know much about how I was nominated as a candidate, because we just got a letter from embassy of Korea that says, “please nominate someone” and rector has nominated someone with related background. (Fellow D, University, Nursing)*

^a^For each quote, the type of affiliated institution and professional background are provided in the parentheses

### Provide enough time for in-class practice and after-class reflection

Most fellows found that incorporating practice a lot of practice contributed significantly to improve educational outcomes. Participants who had experienced other HPE fellowship programs responded that the LJWF-HPE was the most practice-oriented compared to all the others. Limiting formal education to five hours a day was also a positive factor in learning. A fellow suggested shortening the overall duration by increasing the number of hours of instruction each day. The majority, however, was concerned that shortening the duration would deteriorate learning outcomes because of the increased workload per day and reduced time for self-directed learning and self-reflection.

### Selection of fellows

When describing the experience of the application process, most answered that they were recommended by their superiors to apply or just received a notification stating that he/she was nominated as a candidate. In the process, one fellow recalled that the submission of an application was done without being provided the details of the fellowship.

### Participants

#### Heterogeneity of the fellows’ composition

The eight fellows varied in their nationality, major, position, and experience. Because of this heterogeneity, most fellows mentioned that they could broaden their range of ideas and perspectives by sharing experiences from various contexts (Table 5). At this time, the commonality of “Southeast Asian developing countries” was mentioned as a boundary that provided psychological safety, which allowed the fellows to maintain an open attitude.

**Table 5:** Themes and illustrative quotes for the participant domain^a^

Themes	Quotes
Heterogeneity of fellows’ composition	*I came to realize that actual experiences (of the fellows) were fairly different despite our context of seemingly similar under-resourced countries, and even between units in an organization. When talking with other fellows from my university, I realized that we had had different perspectives on the same situation. (Fellow F, University, Pharmacy)*
	*If we have the same background – if all fellows were medical doctors – it might have been possible to go deeper for some topics. With this diversity, facilitators cannot teach in-depth in one area. They rather just have to make overview or cover general theories. (Fellow H, MOH, Medicine)*
Cognitive attributes of participants	*Each of us has different knowledge, and we can help each other. Furthermore, even when one’s theoretical knowledge is limited, it does not matter much because everyone has been involved in various educational activities such as teaching, assessment, and curriculum planning. (Fellow E, MOH, Medicine)*
	*I understand most of the teachings, but I feel difficulty in choosing proper words in English when I want to express my opinion. Listening is okay, but expressing idea is limited (for me). (Fellow H, MOH, Medicine)*
Non-cognitive attributes of participants	*The most important thing is the willingness to share. We can learn while we teach others (Fellow F, University, Pharmacy)*
	*When I did not fully understand lectures, I asked Fellow B, because he explains (lectures) in Khmer. It’s often more comfortable than lectures and makes me confident. (Fellow D, University, Nursing)*
	*Sometimes I wanted to know more details of an area, but I suddenly felt like “other participants might not be that interested as I am”, so I just stayed back and didn’t ask (Fellow E, MOH, Medicine)*
	*I think facilitator E has assumed that we knew the concept (of the lecture), because when he asked us “do you know this?”, we remained silent and just nodded. (Fellow F, University, Pharmacy)*

^a^For each quote, the type of affiliated institution and professional background are provided in the parentheses

*MOH* Ministry of Health

The heterogeneity of the fellows, however, did not always positively contributed to learning. Several fellows pointed out that the diversity often placed limitations on in-depth coverage of some topics, although they acknowledged that the foremost goal of the fellowship was to provide overview and promote general competency in the HPE area.

### Cognitive attributes of the participants

The fellows cited background knowledge, relevant experience, and English communication skills as essential key cognitive attributes of the fellows. Nevertheless, they thought the possession of relevant experience could complement their shortfall in theoretical knowledge on HPE. As for English, fellows emphasized more about the necessity of the ability to express one’s thoughts in class than skills in listening and reading comprehension. Following up on the missed contents was less influenced by English proficiency because most of the learning materials were distributed before or after each class.

### Non-cognitive attributes of the participants

For non-cognitive attributes, the fellows cited cooperative and participatory attitudes as vital to the community because exchanging ideas and experiences could contribute to mutual learning among fellows. Furthermore, fellows could help each other more comfortably as the program progressed and their relationship became more intimate. For some fellows, colleagues from the same country who were willing to explain the contents in their native language, were particularly valued.

Although not explicitly mentioned, fellows’ passivity or hanging back from expressing opinions was also identified as a factor that affected learning. Several fellows attributed missed opportunities for correcting misunderstandings or learning an advanced topic to their unassertive expression of learning needs.

## Discussion

In this study, we explored 12 themes relevant to the process of an international FDP focused on Southeast Asian health professions educators based on O’Sullivan and Irby’s four components of faculty development. The direction of the influence that each factor had on the outcomes was varied by positive, negative, or both, and the findings revealed how the intended outcomes were achieved in this fellowship. Because the themes are connected and inter-related to each other, more implications can be drawn by understanding them in an integrated way.

Developing countries lack educational resources [9, 30], and securing financial support has always been a challenge for any director of FDPs [31]. What this study added, however, is a distinctive feature of the difficulties that developing countries face when encouraging participation in FDPs. In previous studies, financial disincentive was rarely mentioned or treated as a minor factor even when it worked as a barrier [32–34]. In contrast, professors in the fellows’ countries were in a circumstance where one needs to withstand financial losses to attend an FDP. The main reason was that faculty members affiliated with universities frequently own private clinics at the same time, and the fact that health workers in the public sectors in developing countries have dual practices to compensate for low salaries [35]. The opportunity cost of participating in an FDP, therefore, was the income from the private clinics. It prevented faculty members from actively participating in international programs such as the LJWF-HPE, which involves a prolonged leave as well as local FDPs.

One consequence of this systemic difficulty in encouraging FDP participation might be the institutional tendency of relying on a small number of internally motivated and committed faculty members to make an educational improvement. This has both positive and negative sides. On the one hand, this low participation could put an obstacle to the educational reform of an institution because FDPs frequently have an important role to build consensus for change [36]. For instance, a transition to a competency-based medical education should be preceded by the process of defining and communicating key concepts to form an institution-wide cognitive base [37]. From the perspective of an international FDP, on the other hand, those motivated faculty members could be considered as the candidates for potential fellows who will eventually become a critical mass of guiding coalitions, essential for sustaining the organizational change process [38].

Nonetheless, it is questionable whether candidates with the highest motivation and capability from each partnering organization have selected and participated in the LJWF-HPE because many fellows mentioned that they came to be in this fellowship by ‘nomination by their supervisors’ rather than ‘application to open recruitment.’ The theme, “obedience to hierarchy,” is related. In a society that values social hierarchy like the countries of Southeast Asia, individuals in low positions are liable to follow the decisions of those in higher positions rather than adhere to their intentions [39]. When it comes to selection, this hierarchical structure may restrict the pool of potential fellows because of the primary screening of supervisors or administrators. In turn, without clear criteria, the transparency of the selection process could be damaged, and priority may not be given to fellows with sufficient competence and potential as faculty developers.

Despite the potential weakness in the selection process, the key features of effective FDPs identified in the systematic review – applying what fellows learned, relevance and practicality, supportive collegial relationships, using multiple instructional methods, giving feedback, and promoting reflection – were found to be equally valid once the training began [2, 3]. Similarly, as it has been reported that providing learning materials in FDPs has a positive influence on learning [40], fellows stressed the advantages of offering learning materials during the LJWF-HPE. The learning materials helped to review the contents that were not thoroughly understood during class. Additionally, the given resources were valued as high-quality educational materials which could be readily utilized for faculty development activities in the fellows’ countries.

Lastly, the LJWF-HPE’s high heterogeneity of learners and facilitators, arising from the multi-level collaboration, also affected learning. The involvement of twenty-one facilitators not only benefited the fellows as learners by expanding the breadth of the educational contents and perspectives but also helped the fellows as teachers by providing opportunities to experience a variety of facilitation and teaching styles. This is consistent with the existing literature, which highlights increased understanding, widened perspectives, and diverse teaching styles as key advantages of interdisciplinary team teaching [41, 42]. Meanwhile, the effect of the fellows’ heterogeneous composition was less conclusive compared to the existing literature, which regarded the interdisciplinary nature of participants mainly as a strength [43]. Most the fellows appreciated the collective diversity of experience and background, which contributed to broadening their perspectives. At the same time, however, it was pointed out as a limiting factor for learning especially when one wanted focused, in-depth study of a specific topic.

### Implications for faculty development

The themes that emerged in this study provide the following implications for those planning to implement an international FDP in similar settings:

First, appropriate selection strategies should be established. The selection criteria need to include cognitive and non-cognitive attributes emphasized by the fellows. Aligned with adult learning theory [44], appropriate background knowledge and relevant experience will construct the basis of effective learning. More importantly, it should be noted that more values are added when one’s experience is shared with others. Therefore, in the selection process, administrators should seek both English-speaking ability - no less than listening and reading comprehension ability - at the individual level, and an appropriate degree of heterogeneity at a collective level.

Moreover, for effective application and dissemination of learning, which is a higher-level outcome, a fellow’s age (i.e., seniority) and position should not be overlooked, especially in countries of Southeast Asia where power distance is large. Even if the learning outcomes were well achieved during the fellowship period, the possibility of transfer usually decreases if there is only scarce opportunity for application [45]. Therefore, a sequential or complementary approach would be needed, which utilizes both institutional recommendations to enhance ownership and self-determination and criteria-based selection to ensure the desirable attributes of the fellows.

Second, this study demonstrates that the published features of effective faculty development can also be applied to Asian cultures. Although fine adjustments reflecting the differences in cultures and needs are suggested when cross-culturally implementing an FDP [46], it could be attributable to the resemblance of a broader setting, “faculty development.” In practice, the difficulties often originated from communication in English, rather than cultural difference. Participants did not expect the facilitator to have a high level of fluency. Because they were mostly non-native English speakers, English spoken by facilitators would be advantageous when it is easy, concise, and understandable.

Third, the formation of a safe learning environment is vital. One notable finding of this study is that the passive attitudes of the fellows were commonly witnessed in ways that negatively affected learning. In Asian culture, it is prevalent among learners to avoid questioning or revealing personal thoughts [47]. The silence, − and sometimes even their smile – however, does not necessarily imply agreement with other people, including teachers [48]. If interaction and discussion of ideas are indispensable factors for a workshop [49], the necessity of a safe learning environment that can facilitate the exchange of opinions and mutual feedback should be given more attention especially in a cultural context such as those of Asian countries.

### Strengths and limitations

The study has some strengths and limitations. One strength is that it used a conceptual framework, a hallmark of clarification studies [17]. Unlike other curriculum models in general [50, 51], this framework was developed specifically for faculty development research. Therefore, with this framework, we could comprehensively and efficiently identify the key themes for our fellowship. Another strength is that this study explored the experiences and perspectives of health professional educators from non-English speaking, Southeast Asian developing countries, who have been marginalized so far. By focusing on the perception of the fellows, it was possible to “give voice” to the participants, which is an important function of qualitative research [52].

On the other hand, one of the limits of this study were that it was based on the experience of one program and its participants. Nevertheless, the fact that the LJWF-HPE demonstrated comparable outcomes to the SICME (see Additional file 2) implies that these factors may not be unique to LJWF-HPE. In addition, many common features can be found between this study’s findings and those of the previous FDP studies. Second, although a qualitative study may not require a large sample [53], the number of participants in this study is relatively small. Therefore, to maximize the credibility of the data collection, all fellowship participants were interviewed both individually and in a group to reach data saturation. Furthermore, there are studies that report meaningful results using data collected from participants of similar size [31, 54–56]. Third, because the data were collected when the fellowship was in progress, the findings might be more focused on learning during the program rather than transfer after completing the program. In addition, the four predetermined components of the conceptual framework used in this study might have resulted in a failure to uncover other factors that contributed to the fellowship outcomes. Further research is needed to determine what kinds of additional factors exist regarding the transfer of learning in their workplace.

## Conclusion

This study investigated which factors of an international FDP, aimed at educational capacity building of health professional educators in Southeast Asia, had an influence on achieving the intended outcomes. Overall, the themes identified were well fitted with O’Sullivan and Irby’s four components of faculty development. A closer look reveals that there are factors that deserve specific attention in a Southeast Asian context including appropriate selection strategies, a safe learning environment, and English communication. Although the specific settings of the LJWF-HPE might limit the transferability of the findings, these themes can be used to develop tailored strategies to overcome the frequent challenges of international collaboration for HPE.

## Additional files

Additional file 1: Process-oriented evaluation 03. Interview agendas for participants. A guide for semi-structured interviews for LJWF-HPE. (DOCX 20 kb)

Additional file 2: Process-oriented evaluation 03. Quantitative evaluation of level 1 (reaction) and level 2 (learning) of the Kirkpatrick model. Comparison of the evaluation results between two programs, which shows the LJWF-HPE demonstrated comparable outcomes to the SICME. (DOCX 22 kb)

## Abbreviations

FDP: Faculty development program
HPE: Health professions education
KOFIH: Korea Foundation for International Healthcare
LJWF-HPE: Lee Jong-Wook Fellowship for Health Professional Education
MOH: Ministry of Health
SICME: Seoul Intensive Course for Medical Educators
UHS: University of Health Sciences
