# Supplementary material for: Process-oriented evaluation of an international faculty development program for Asian developing countries: a qualitative study
Source: BMC Med Educ. 2017 Dec 21;17:260. doi: 10.1186/s12909-017-1101-2 (PMC5740877; doi:10.1186/s12909-017-1101-2)
Supplement: Supplementary file 1 — Process-oriented evaluation 03. Interview agendas for participants. A guide for semi-structured interviews for LJWF-HPE. (DOCX 20 kb) [file 12909_2017_1101_MOESM1_ESM.docx]

**Additional file 1**

**Interview agendas for participants**

# (1) Before fellowship

1. How did you start the role as a teacher? (I)
2. If you have, please describe your role model or mentor as a health professions educator. (I)
3. What types of feedback have you received from your colleagues or students about your teaching practice? (I)
4. How and why did you decide to participate in this fellowship? (I)
5. How did your colleagues react to your participation in this fellowship? (I)
6. To what extent does your institution support faculty members’ educational improvement? (G)

# (2) During fellowship

1. Which **factors** contributed to satisfaction with the module (or fellowship)? (G)
2. What is your strength or weakness as a **fellow**? (I)
3. How did **your English competency** influence your learning? (I)
4. Which **facilitator** delivered most effectively? Which characteristics contributed to the effectiveness? (G)
5. How did the interaction or relationship with **facilitators** change as the program proceeds? (G)
6. What is your opinion about having an **English translator**? (G)
7. How did the other **fellows** influence your learning? (I)

# (3) After fellowship

1. How did you utilize your **free-time**? (I, G)
2. How did you use the **materials** provided (soft copy, hard copy)? (G)
3. How have you **developed** as a health professions educator after the module (or fellowship)? (G)
4. **What else did you learn** during the fellowship beside the previously described objectives of each module? (I)
5. What do you want to **apply** the most among the contents covered in the fellowship? (G)
6. What kind of **support do you need** to apply what you learned in your country/institution? (G)

I: Questions primarily for individual interview, G: Questions primarily for group interview
