# Supplementary material for: Process-oriented evaluation of an international faculty development program for Asian developing countries: a qualitative study
Source: BMC Med Educ. 2017 Dec 21;17:260. doi: 10.1186/s12909-017-1101-2 (PMC5740877; doi:10.1186/s12909-017-1101-2)
Supplement: Supplementary file 2 — Process-oriented evaluation 03. Quantitative evaluation of level 1 (reaction) and level 2 (learning) of the Kirkpatrick model. Comparison of the evaluation results between two programs, which shows the LJWF-HPE demonstrated comparable outcomes to the SICME. (DOCX 22 kb) [file 12909_2017_1101_MOESM2_ESM.docx]

**Additional file 2**

**Quantitative evaluation of level 1 (reaction) and level 2 (learning) of the Kirkpatrick model**

| Levels | Questions | SICME ^a^ | LJWF-HPE ^a^ | *p*-value ^b^ |
| --- | --- | --- | --- | --- |
| Level 1 (reaction) | 1. Contents ^c^ | 3.68±0.47 | 3.66±0.48 | 1.000 |
|  | 2. Facilitators ^c^ | 3.72±0.45 | 3.51±0.53 | 0.083 |
|  | 3. Facilities ^c^ | 3.63±0.48 | 3.49±0.57 | 0.173 |
|  | 4. Materials ^c^ | 3.63±0.48 | 3.58±0.50 | 0.515 |
|  | 5. Schedule ^c^ | 3.51±0.53 | 3.36±0.60 | 0.237 |
|  | 6. Relevance to one’s needs and interests ^c^ | 3.71±0.46 | 3.55±0.50 | 0.083 |
|  | 7. Proportion of activities to lecture ^c^ | 3.52±0.52 | 3.34±0.64 | 0.274 |
|  | 8. Achievement of stated goals and objectives ^c^ | 3.55±0.52 | 3.41±0.52 | 0.237 |
|  | 9. Help to do one’s job better ^c^ | 3.66±0.48 | 3.45±0.59 | 0.315 |
|  | 10. Overall satisfaction ^c^ | 3.57±0.50 | 3.39±0.56 | 0.237 |
| Level 2 (learning) | 11. Improvement of knowledge ^d^ | 3.45±0.50 | 3.28±0.53 | 0.034 |
|  | 12. Improvement of skills ^d^ | 3.31±0.46 | 3.13±0.58 | 0.068 |
|  | 13. Improvement of attitudes ^d^ | 3.53±0.50 | 3.28±0.50 | 0.002 |
|  | 14. Overall competency (Pre-module) ^e^ | 1.76±0.78 | 1.04±0.52 | < 0.001 |
|  | 14. Overall competency (Post-module) ^e^ | 3.46±0.69 | 3.47±0.74 | 1.000 |

^a^ Average of all modules, Mean±SD ^b^ Independent sample Mann-Whitney U test; ^c^ Rating scale: 1 (Very poor) – 4 (Very good); ^d^ Rating scale: 1 (not improved at all)—4 (very much improved); ^e^ Rating Scale: 1 (Novice/Knows what), 2 (Advanced beginner/Knows how), 3 (Competent/Shows how), 4 (Proficient/Does), and 5 (Expert/Mastery)
